# The Effects of Codon Context on *In Vivo* Translation Speed

**DOI:** 10.1371/journal.pgen.1004392

**Published:** 2014-06-05

**Authors:** Fabienne F. V. Chevance, Soazig Le Guyon, Kelly T. Hughes

**Affiliations:** 1 Department of Biology, University of Utah, Salt Lake City, Utah, United States of America; 2 Department of Microbiology, Tumor and Cell Biology, Karolinska Institute, Stockholm, Sweden; Universidad de Sevilla, Spain

## Abstract

We developed a bacterial genetic system based on translation of the *his* operon leader peptide gene to determine the relative speed at which the ribosome reads single or multiple codons *in vivo*. Low frequency effects of so-called “silent” codon changes and codon neighbor (context) effects could be measured using this assay. An advantage of this system is that translation speed is unaffected by the primary sequence of the His leader peptide. We show that the apparent speed at which ribosomes translate synonymous codons can vary substantially even for synonymous codons read by the same tRNA species. Assaying translation through codon pairs for the 5′- and 3′- side positioning of the 64 codons relative to a specific codon revealed that the codon-pair orientation significantly affected *in vivo* translation speed. Codon pairs with rare arginine codons and successive proline codons were among the slowest codon pairs translated *in vivo*. This system allowed us to determine the effects of different factors on *in vivo* translation speed including Shine-Dalgarno sequence, rate of dipeptide bond formation, codon context, and charged tRNA levels.

## Author Summary

The central dogma of molecular biology states that DNA is transcribed into RNA, which is translated into protein. The process of translation from messenger RNA (mRNA) into protein by the ribosome is a complicated process involving transfer RNA intermediates with attached amino acids that must recognize 3 base codons in the mRNA sequence to be translated with the correct amino acid. With 4 bases to code for 20 proteins the genetic code is redundant with 61 coding codons and three stop codons. A given amino acid might be coded by one, two, three, four or six different codons. We developed a system that measures the speed of the ribosome *in vivo* during translation. Using this system, we show that the ability of a given codon to be translated is dependent on its neighboring codons, the codon context effect.

## Introduction

Synonymous mutations are DNA (or mRNA) sequence changes that do not affect the protein amino acid sequence. It is now established that synonymous codon usage is neither random nor neutral [1]–[6]. Different organisms use different codons at low frequencies including so-called rare codons. The expression of foreign proteins in *E. coli* is dramatically affected by the presence or absence of rare codons in the coding sequence. Manipulation of coding sequences for foreign proteins to coincide with preferred codon usage in the host organism is often essential for the expression of heterologous proteins.

By 1980, direct evidence that mRNA sequence outside the codon (context) can affect the efficiency of translation was presented. The ability of a nonsense suppressor tRNA to recognize a stop codon was dramatically affected by the adjacent codon [7]. This effect was in the context of recognition of three bases by the tRNA and not four [8]. Similar results were later observed with suppression of missense mutations [9]–[11]. These studies provided *in vivo* genetic evidence for context effects on translation.

Codon usage has also demonstrated differences in translational accuracy *in vivo* [12]–[14]. Genes expressed to different degrees evolve at different rates [15]–[18]. Synonymous codon changes to either non-rare or rare codons are known to affect translation rates in *E. coli*; whereas silent mutations that remove or generate rare codons have been shown to increase or decrease mRNA stability [19]–[20]. A single synonymous serine codon change confers the ability of *Bacillus subtilis* to form biofilms [21]. Synonymous codons can have a profound effect at translation initiation on mRNA folding to affect translation efficiency [22]. Thus ribosome pausing can affect RNA exposure to endonuclease attack [23].

Varying the speed of translation has been implicated as important in controlling the efficiency at which proteins are translated [24] and in facilitating the proper folding of protein as they are translated [25]. Any effect of synonymous codon changes on translation speed would likely be subject to strong evolutionary pressures. Recent work using ribosome profiling demonstrated that synonymous changes to a coding sequence that is more identical to the Shine-Dalgarno sequence [26] results in translational pausing in *E. coli* [27]. Unexpectedly, rare codons did not lead to slow translation under nutrient rich conditions by the ribosome profiling method [27].

**Figure 1:**
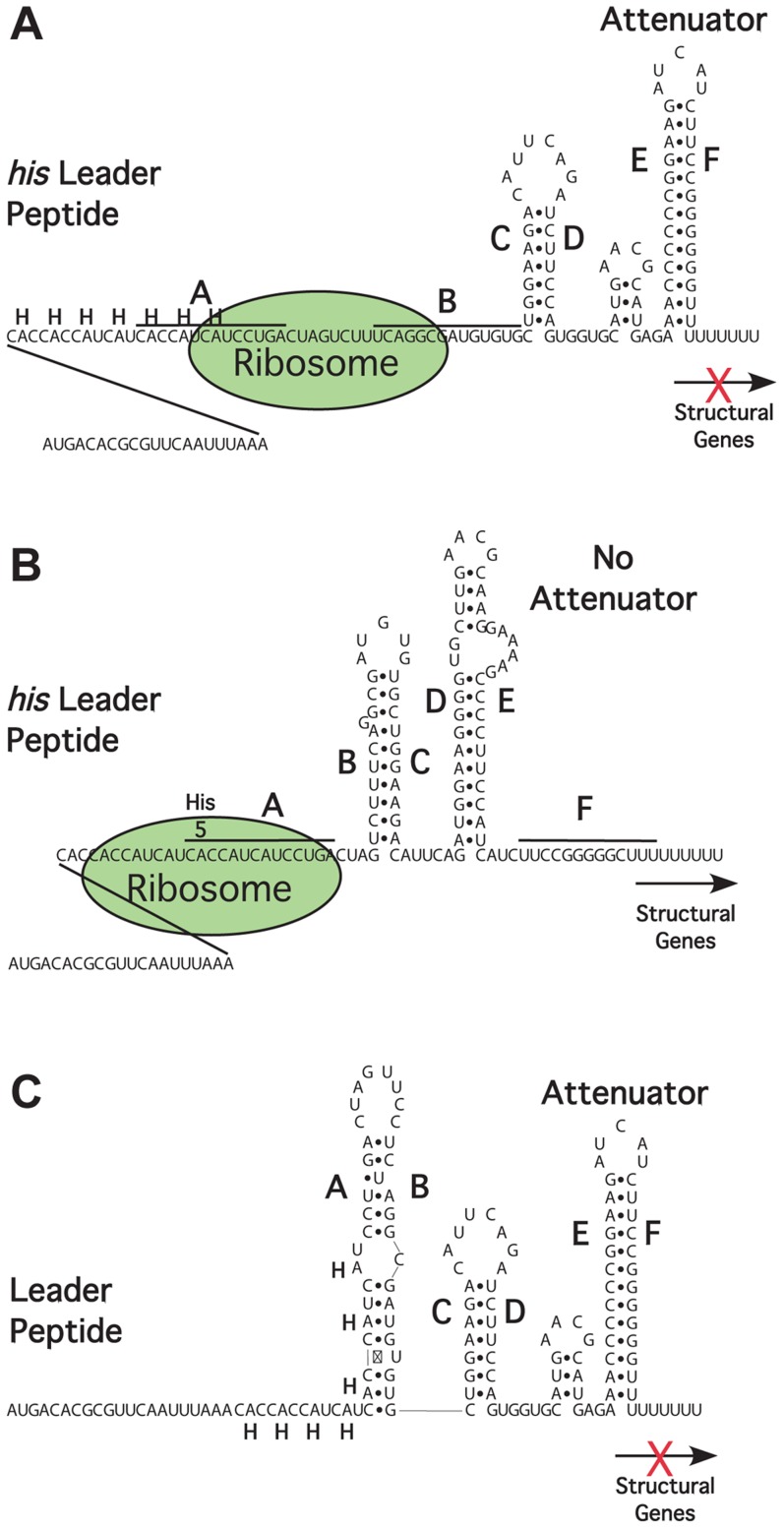
Attenuation mechanism for the regulation of the histidine biosynthetic operon of *Salmonella enterica* [28]. The 5' regulatory region of the *his* operon encodes a 16 amino acid leader peptide, including 7 consecutive His codons, followed by a transcription terminator. **A**. Under conditions where histidyl-tRNA levels are high ribosomal translation proceeds through the leader peptide to its stop codon. This results in the formation of the E:F attenuator stem-loop and inhibition of further transcription into the *his* structural genes. **B**. Under conditions of limited histidyl-tRNA, the translation through the His codons is slowed and the stalled ribosome allows for the formation of an alternative RNA secondary structure that occludes attenuator formation. Inhibition of attenuator formation allows RNA polymerase to continue transcription into the *his* operon structural genes. **C**. Under conditions where transcription from the *his* promoter is not coupled to translation the E:F attenuator is predicted to form and inhibition of further transcription into the *his* structural genes.

In order to monitor context effects on translational kinetics in a more general manner and under entirely *in vivo* conditions, we developed a genetic assay in which ribosomal speed could be measured independently of the stability of the mRNA transcript or the translated protein product. This assay exploits the peculiar kinetic features of the histidine operon transcription attenuation mechanism [28]. Transcription initiating at the *his* operon promoter can either stop prematurely at a terminator site (the attenuator) or proceed into the structural genes. The decision to continue transcription into the *his* structural genes depends on the rate of translation of a peptide encoded in the leader portion of the mRNA, which comprises a 16-codon stretch including 7 histidine codons in a row (Figure 1). When the leader mRNA is efficiently translated in the presence of excess histidinyl-tRNA (His-tRNA), the terminator structure forms immediately downstream from the leader peptide (E:F hairpin in Figure 1A) and prevents transcription from entering the structural genes of the operon. In contrast, a slowdown of the ribosome, consequent to a decline in the His-tRNA concentration, causes the nascent RNA to adopt an alternative conformation (B:C and D:E hairpins, Figure 1B) that allows transcriptional read-through at the attenuator site. Continued transcription into structural genes of the *his* operon thus requires translation of the *his* leader sequence. The default secondary structure of an untranslated, 5′ *his* operator RNA region is the E-F attenuator stem loop because A is free to pair with B, which prevents formation of the B-C stem-loop (Figure 1C).

Other protocols have been developed to measure translation speed such as the "speedometer" assay developed by Drs. James Curran & Michael Yarus in which translational frameshifting dependent on slow decoding of an in frame codon is used to assess translation rate [29]. This very sensitive system has been used to compare the rates of translation of many codons. An advantage of the system described in this manuscript is that it is wholly unaffected by primary sequence since the structure of the His leader peptide is irrelevant to the assay.

## Results

### Use of the *his* operon attenuation system to measure the *in vivo* speed of ribosomal translation

A *lac* operon was fused to the histidine promoter-operator control system by insertion of a Mud-*lac* transcriptional reporter element into the *hisD* structural gene [30]. Accordingly, the expression of the histidine operon is readily assessed, enzymatically, measuring β-galactosidase (β-gal) activity, and visually (qualitatively) on lactose indicator media such as Tetrazolium-lactose, MacConkey-lactose or standard rich medium (LB) containing X-gal. In either rich or minimal medium, the *hisD-lac* is expressed at a low level and this level does not change appreciably upon addition of histidine to the medium. De-attenuation of the *his* biosynthetic operon occurs in a strain unable to synthesize histidine, such as a histidine auxotroph with limiting exogenous histidine.

### Forced pausing in the *his* leader sequence results in loss of attenuation

**Figure 2:**
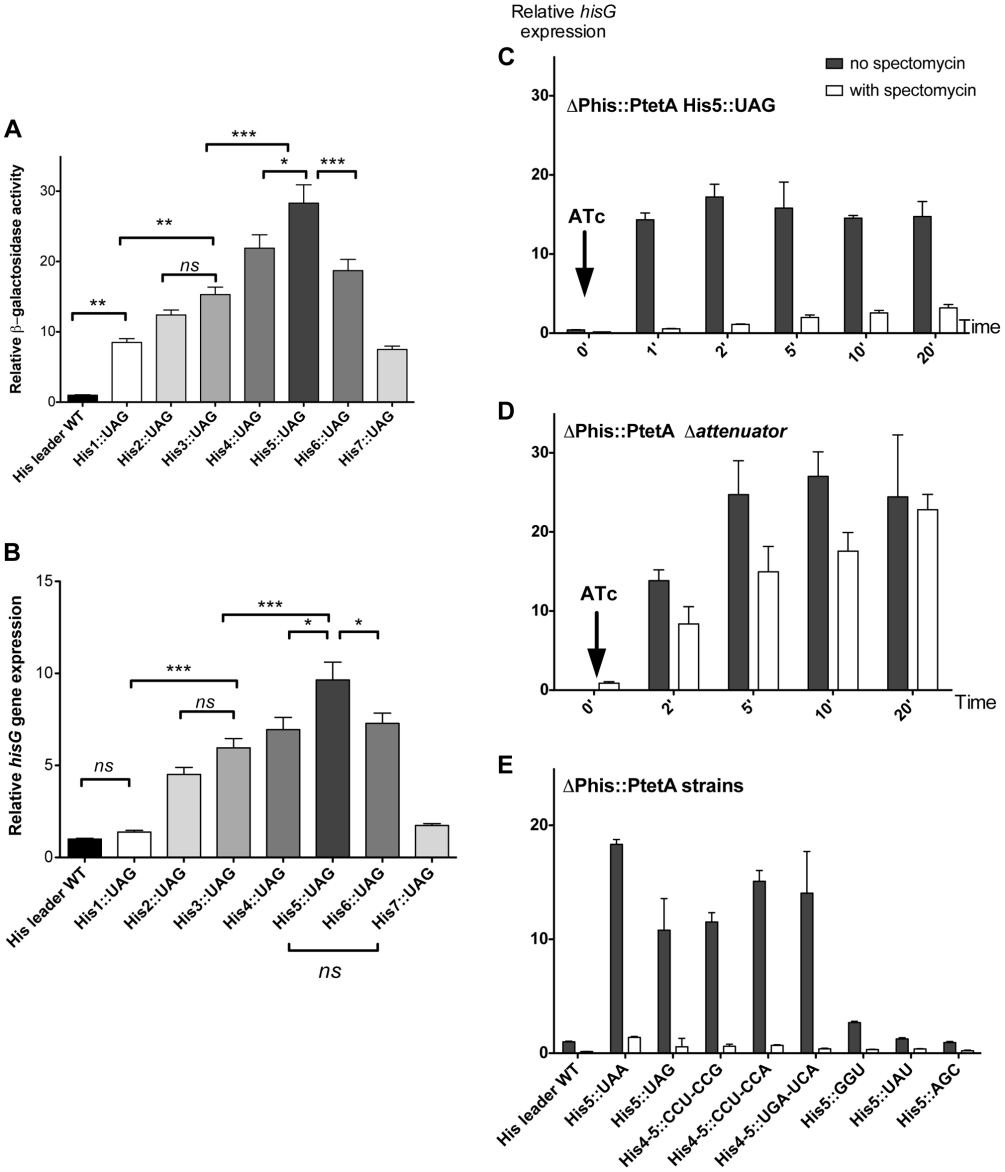
Effect of codon substitutions in the *his* leader region on derepression of *his* operon transcription. (**A** & **B**) Each of the seven His codons in the His leader peptide were replaced with UAG stop codons and the effect of *hisD-lac* (**A**) or *hisG* transcription was determined by either β-galactosidase assay for *hisD-lac* (**A**) or quantitative real time-PCR for *hisG* mRNA (**B**). Errors bars represent the standard deviation of the means. One-way analysis of variance followed by Turkey test showed which data set were statistically different (*ns* (not significant); * (P<0.05); ** (P<0.01); *** (P<0.001)). For panel A, means were significantly different at least at 95% confidence interval levels except for the means of His1::UAG vs His2::UAG; His1::UAG vs His7::UAG; His2::UAG vs His3::UAG; His2::UAG vs His7::UAG; His3::UAG vs His6::UAG; His4::UAG vs His6::UAG), that were not significantly different. For panel B, means were significantly different at least at 95% confidence interval levels except for the means of His leader WT vs His1::UAG; His leader WT vs His7::UAG; His1::UAG vs His7::UAG; His2::UAG vs His3::UAG; His3::UAG vs His4::UAG; His3::UAG vs His6::UAG and His4::UAG vs His6::UAG that were not significantly different. (**C**, **D** & **E**) The mRNA levels of *hisG* were determined using quantitative RT-PCR. For all the strains analyzed, the natural *his* promoter was replaced with the P*_tetA_* promoter, to allow induction of *his* operon transcription with addition of the P*_tetA_* inducer anhydrotetracycline (ATc). Strains were grown to OD ∼0.3, at which time spectinomycin was added to inhibit translation. When OD reached 0.4, anhydrotetracycline (ATc) was added. **C**: mRNA from P*_tetA_* His5::UAG was extracted at different time points after induction of *his* operon transcription with and without added spectinomycin to inhibit translation and *hisG* mRNA levels were determined by qRT-PCR. **D**: mRNA from the *his* E-F attenuator stem-loop deletion strain under control of P*_tetA_* was collected at different time points after induction of *his* operon transcription with and without added spectinomycin to inhibit translation and *hisG* mRNA levels were determined by qRT-PCR. **E**: The *hisG* mRNA levels were determined by qRT-PCR from different His5 single codon substitution and His4-His5 double codon substitution mutants at one-minute time points after *his* operon induction from P*_tetA_* by ATc in the presence and absence of spectinomycin.

As a proof-of-principle, each of the seven His codons in the *his* leader peptide was separately replaced with a UAG stop codon. We predicted that the ribosome would stall at these stop codon positions and result in loss of attenuation as shown in Figure 1B. This was tested by assaying β-galactosidase from the *hisD-lac* transcriptional fusion and measuring *hisG* mRNA levels by quantitative RT-PCR. The results of either assay were comparable as shown in Figure 2 (A & B). Thus, as predicted by the *his* attenuation model, insertion of a UAG stop codon at any of the seven His codon positions resulted in reduction of attenuation and higher levels of the *his* operon expression (Figure 2, A & B). Stalling at central positions His4 through His6 resulted in the highest level of expression.

Since sequence changes in the leader RNA could in principle affect its folding properties, and thus the behavior of attenuation, it was important to verify that *his* operon de-attenuation in strains with the UAG His-leader substitutions required that the RNA leader be translated. To do this, the *his* operon promoter was replaced with the inducible *tetA* (P*_tetA_*) promoter from transposon Tn*10* and *hisG* mRNA levels quantified by RT-PCR following P*_tetA_* activation with anhydro-tetracycline (ATc). This assay reproduced the effects of the His5-UAG mutant previously observed with *lac* measurement (Figure 2C). However, when the P*_tetA_* promoter was activated in the absence of protein synthesis (spectinomycin-treated cells), *hisG* mRNA levels remained low (Figure 2C) suggesting that transcription terminated at the attenuator site. Indeed, removal of E-F attenuator sequence essentially abolished this effect and restored high *hisG* mRNA levels in the absence of translation (Figure 2D). These results are consistent with the early finding that a nonsense mutation at amino acid position 5 in the *his* leader peptide gene result in uninducible operon expression and a His^−^ phenotype [31]–[32]. Thus, we validated the hypothesis that transcription of the *his* structural genes requires translation of the leader peptide gene and ribosome stalling at the 7 consecutive His codons of this gene sequence. Overall, these tests support the conclusion that the *his* regulatory system can be used to measure ribosome stalling during mRNA translation *in vivo*.

### Effect of randomization of the His5 codon in the *his* leader peptide gene on attenuation

**Figure 3:**
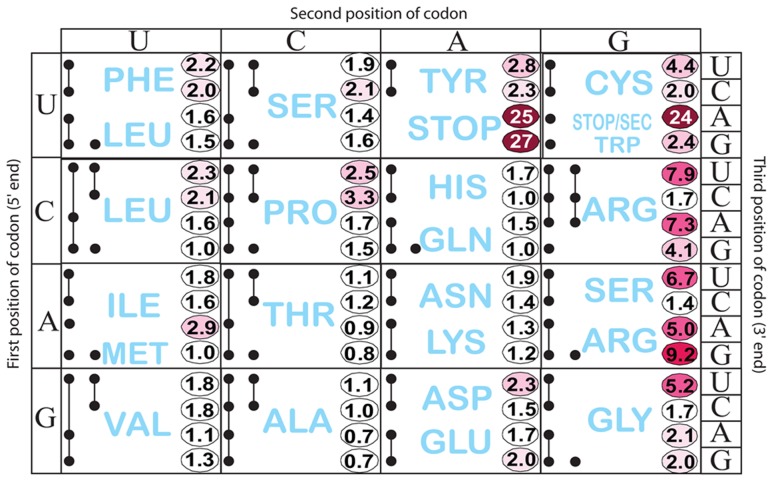
Histidine operon expression with His5 of the leader substituted by all 64 codons. All constructs carry a *hisD-lac* operon fusion that places the *lac* operon under control of the *his* operon promoter-attenuator regulatory system. The number in each box is the β-galactosidase activity of each mutant construct divided by the activity of the wild-type construct that contains the CAC His codon at His5. This figure also shows all tRNA species represented by a single or multiple solid black circles connected by lines according to Glenn Bjork [61]. If a tRNA reads a single codon, it is represented by a single black dot (ie. UGG Trp). If a given tRNA reads multiple codons the codons it reads are represented by solid black dots connected by lines.

We tested the effect of changing His codons of the *his* leader peptide gene. Having observed the greatest ribosome-stalling effects with the UAG at position His5, we first randomized codon His5. This was done in a strain with a *hisD-lac* operon fusion so that the effects on *his* expression could be immediately evaluated by examining the color of colonies on lactose indicator medium. A set of 64 constructs, each with a different codon at His5 in the *his* leader was constructed in the *hisD-lac* background and visualized on MacConkey-lactose indicator medium. Loss of attenuation was quantified by β-galactosidase assay (Figure 3 and Figure S1A). Figure 3 shows the level of *hisD-lac* expression with all 64 codons present at the His5 position in the *his* leader peptide gene relative to the wild-type CAC codon. The data are presented using the codon chart that also includes all tRNA species represented by black dots. Two or more dots connected by a line indicate one or more tRNA gene products that read more than one codon.

As expected, stop codons at position His5 showed the biggest effect causing 24- to 27-fold de-repression of *hisD-lac* transcription. Interestingly, the highest degree of stalling for a non-stop codon occurred with the rare AGG Arg codon. The other two rare Arg codons, CGA and AGA also exhibited a high degree of stalling. The AUA (Ile) rare codon exhibited intermediate stalling, but CUA (Leu) did not. This is consistent with ribosome profiling results showing that rare codons did not lead to slow translation under nutrient rich conditions unless changes result in a coding sequence that is more identical to the Shine-Dalgarno sequence [27]. Three other codons CGU (Arg), AGU (Ser) and GGU (Gly), although not rare, exhibited a high degree of stalling with GGU resulting in a significant change to a more Shine-Dalgarno sequence. Also of significance is the variation in *hisD-lac* expression seen for different codons for a single amino acid (synonymous changes). We were concerned that sequence changes in the His4-His5 positions resulting in increased or decreased free energies for AB stem-loop formation could account for differences in de-attenuation levels by increasing or decreasing the probability of EF attenuator formation. In order to rule out possible effects of mRNA secondary structure on *his* attenuation, *hisG* mRNA levels for 5 different His5 variants (including UAA and UAG stop codon positions) and 3 highly de-attenuated double substitutions at His4-His5 (discussed below) were expressed from P*_tetA_* in the presence and absence of spectinomycin (i.e., with and without translation of the *his* leader peptide gene). Blocking translation reduced *hisG* mRNA levels confirming the dependence of *hisG* transcription on leader peptide gene translation (Figure 2E). In addition, we found that changes in the free energies of AB stem-loop formation by sequence changes in His4 and His5 did not correspond to changes in de-attenuation levels. Thus, the *his* leader assay system can detect and quantify single codon context effects on mRNA translation by the ribosome.

For most coding pairs differing by U/C in the third position (NNU/C), the codon with C in the 3^rd^ position is translated faster than NNU codons. This in agreement with previous studies by Thomas et al. [33], which showed that specific Phe and Leu tRNAs react faster with NNC than with NNU, and by Curran and Yarus [29], which showed that specific Phe, Leu, Tyr, His, Cys and Arg tRNAs translate NNC faster than NNU. We found that the bias of NNC over NNU was most prominent when only one tRNA species read the NNU/C codon and the third position codon-anticodon pair is G. In the case of the CGU/C codons there are two tRNA species with inosine (I) in the anticodon that pairs with the U or C in the 3^rd^ codon position. The I-containing anti-codon:codon pairing also shows a strong bias for pairing with G over U. These results are consistent with differences in stabilities of tRNA:tRNA complexes based on G:C versus G:U pairing [34]. Since there are higher affinities for tRNA with a G:C or I:C 3^rd^ position pair relative to a G:U or I:U 3^rd^ position pair, our results suggest that a stronger anti-codon:codon interaction results in faster *in vivo* translation of particular codons.

### 5′ and 3′ effects of codon context on translation of the UCA codon by SerT tRNA

Once we determined that *his* de-attenuation provided a sensitive indicator for ribosome stalling at the His5 position, we decided to use this system to analyze the effect of codon context on the apparent *in vivo* translational speed. We chose to study the effects of codon context on the reading of the UCA codon knowing that UCA is recognized by a single tRNA species, SerT, and that UCA is not a significant Shine-Dalgarno sequence (that could interact with the 16s anti-SD sequence: 5′-CACCUCCU-3′). In addition, the lab has previously worked with a conditional-lethal mutation in *serT* [35]. In order to study 5′- and 3′-effects separately, two sets of constructs were made carrying the UCA codon at either His5 or His4 positions. For 5′-effects (UCA at His5), the His4 triplet was randomized with all 64 codons. For 3′-effects (UCA at His4), the His5 triplet was randomized with all 64 codons.

**Figure 4:**
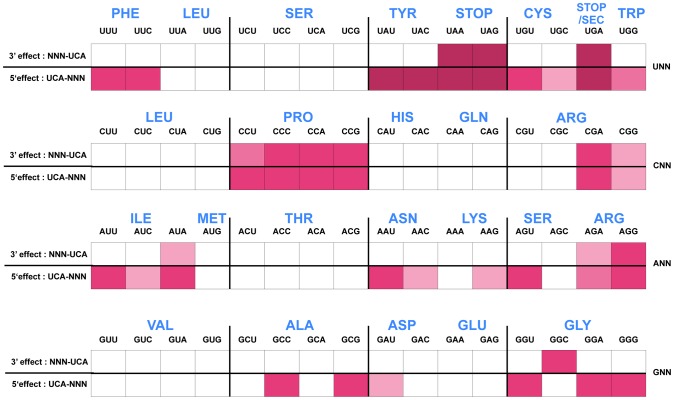
Histidine operon expression phenotypes of UCA-NNN and NNN-UCA substitutions at His4-His5 of the His leader peptide. The *his* operon leader peptide was altered with either NNN-UCA (3′-effect) substituted at positions His4-His5, or UCA-NNN (5′-effect) substituted at positions His4-His5. All constructs carry a *hisD-lac* operon fusion that places the *lac* operon under control of the *his* operon promoter-attenuator regulatory system. Levels of *hisD-lac* transcription are qualitative as determined by a color phenotype on MacConkey lactose indicator medium where white is Lac^−^ and the redder the Lac^+^ colonies, the greater the levels of β-galactosidase expressed from *hisD-lac*.

The His4::UCA His5::NNN and His4::NNN His5::UCA effects on *his* operon transcription, as measured by the *hisD-lac* operon fusion expression on Mac-Lac indicator medium, are presented in Figure 4. High level *lac* expression indicate reduced attenuation resulting in a dark red color on MacConkey-lactose (Mac-Lac) indicator plates; low level *lac* expression results in a white colony phenotype and intermediate *lac* levels produce a range in MacConkey colony phenotypes from light pink to red in color. The colony phenotypes are represented by the color for each codon pair as shown in Figure 4. High levels of β-gal activity (dark red color on Mac-Lac indicator plates) are consistent with a prolonged stalling of the ribosome during translation of the leader mRNA. The data is presented to facilitate comparison of 5′ and 3′ effects of the specific NNN-UCA and UCA-NNN codon pairs. As expected, insertion of stop codons resulted in the greatest effects regardless of whether 5′ or 3′ to a UCA codon. Interestingly, other codons displayed a strong stalling effect as well - in particular the tyrosine (Tyr) codons UAU/C when placed on the 3′ side of UCA. Both the UCA-UAU and UCA-UAC combinations at His4-His5 resulted in de-attenuation levels comparable to those observed with stop codons. However, when the UCA-UAU/C pairs were reversed to UAU/C-UCA, there was no significant loss of attenuation (white colony color on Mac-Lac), suggesting that codon context rather than limiting Tyr-tRNA accounted for the loss of attenuation in the UCA-UAU/C constructs.

**Figure 5:**
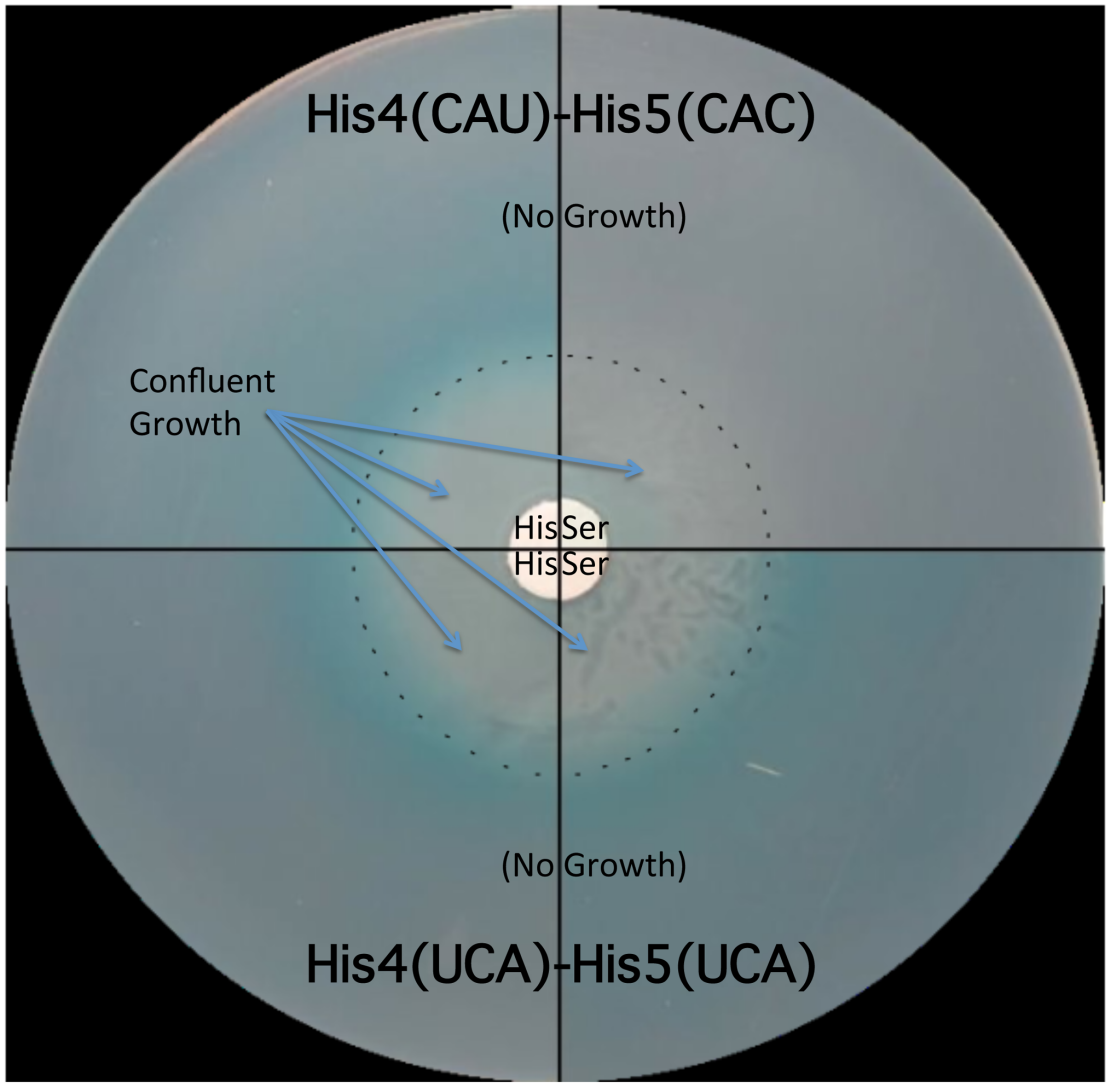
The His leader with UCA(Ser)-UCA(Ser) at His4-His5 responds to either histidine or serine starvation for loss of attenuation. Two strains were tested for the effects of either histidine or serine starvation on loss of *his* operon attenuation: a strain with a wild type His leader with His codons at His4-His5 (CAU-CAC) and a strain with UCA(Ser)-UCA(Ser) codons at His4-His5. Both strains carried a *hisD-lac* operon fusion and a *serB*::Tn*10* insertion resulting in auxotrophy for both histidine and serine. A 0.1 ml portion containing 10^8^ cells for each strain was plated on minimal glucose X-gal medium supplemented with either serine or histidine. For plates supplemented with histidine, serine was added to a filter disc placed at the center of the plate. For plates supplemented with serine, histidine was added to a filter disc placed at the center of the plate. The plates were incubated overnight at 37°C. Confluent growth occurred near the filter discs containing histidine or serine supplements and growth was inhibited as the concentration of supplements became limiting as indicated by the dashed line. For the strain with histidine codons at His4 and His5 of the *his* leader peptide starvation for histidine resulted in de-attenuation and expression of the *hisD-lac* operon fusion indicated by the production of the X-gal blue color at the position in the plates where the cells are starved for histidine, while starvation for serine did not result in de-attenuation of *his* operon expression (no blue color at the position in the plates where the cells are starved for serine). For the strain with serine codons at His4 and His5 of the *his* leader peptide, starvation for either serine or histidine resulted in de-attenuation and expression of the *hisD-lac* operon fusion.

The strong effect of the UAG codon substitutions in the *his* leader peptide gene on loss of attenuation suggests that the *his* system can be modified to respond to limiting tRNAs corresponding to other amino acids besides histidine. We tested this by examining whether the double substitution of His4-His5 from CAU(His)-CAC(His) to UCA(Ser)-UCA(Ser) might respond to serine starvation. A *serB*::Tn*10* insertion was combined with the *hisD-lac* reporter insertion (*hisD*::MudJ) resulting in a double auxotrophic requirement for both histidine and serine. The strains were plated on minimal glucose medium supplemented with either serine or histidine and X-Gal as an indicator of *hisD-lac* transcription (Figure 5). For plates supplemented with serine, histidine was added to a filter disc placed at the center of the plate. Conversely, for those plates supplemented with histidine, serine was added to a filter disc placed at the center of the plate. The cells grew near the filter discs, but growth diminished at greater distances where they were starved for either histidine or serine (Figure 5). For the strain with the wild-type His leader sequence, starvation for histidine de-attenuated *hisD-lac* transcription is indicated by the blue color at the zone of transition from confluent growth to no growth on the plate while starvation for serine did not induce *hisD-lac* transcription. For the strain carrying the UCA(Ser)-UCA(Ser) serine codon pair at the His4-His5 positions in the His leader peptide, starvation for either histidine or serine resulted in induction of *hisD-lac* transcription when the cells were starved for either serine or histidine.

### Formation of the Pro-Pro peptide bond is a slow process *in vivo*

It has been reported that the addition of a proline residue, the sole N-alkylamino acid (imino acid) in the genetic code, during ribosome translation occurs at a slower rate than insertion of other amino acids due to the unusual nature of the cyclic proline residue that constrains the protein secondary structure [36]. When single proline codons are placed in the *his* leader system in context with His codons or with UCA, some loss of attenuation was observed, but the effect on de-attenuation was not as dramatic as observed with a stop or rare arginine codons (Figures 2 & 3).

In a screen for codon sequences that induce ribosome stalling, Tanner et al. [37] identified amino acid sequences ending in di-proline residues that caused ribosome stalling within a coding region, *in vivo*. We reasoned that successive proline codons could have a synergistic effect on translation speed. The recent discovery of a translation elongation factor, EF-P, which is required for the efficient translation of consecutive proline residues within a coding sequence supports this hypothesis [38]. Thus, we tested for the effects of consecutive proline codons at His4-His5 in the *his* leader system on loss of attenuation.

**Figure 6:**
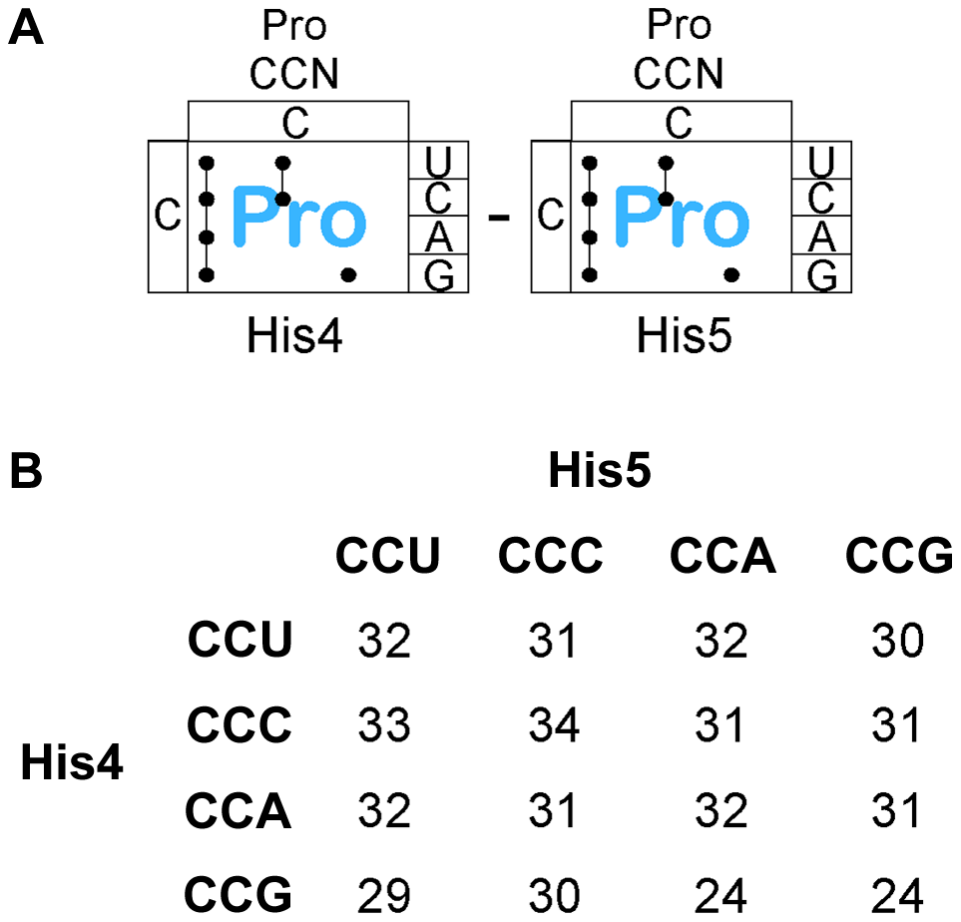
Expression of the *his* operon for specific proline codons at His4-His5 of the His leader on ribosome stalling. **A**. There are three Proline tRNA species in *Salmonella* that read the CCN – CCN pairs placed at the His4-His5 codon positions in the His leader. **B**. The levels of *hisD-lac* transcription for all strains with the His4-His5 codon positions in the His leader substituted with the 16 possible Pro-Pro codon pairs were determined by β-galactosidase assay and the fold depresession compared to wild-type *hisD-lac* transcription was determined.

There are 4 proline codons (Figure 6A), CCN. All 16 CCN-CCN codons pairs were constructed at His4-His5 and their effect on loss of attenuation and resulting *hisD-lac* expression was determined. As shown in Figure 6B, all 16 Pro-Pro codons at His4-His5 resulted in loss of attenuation and high level *hisD-lac* expression similar to that observed with stop codons (see Figure S1B for specific β-Gal activities). This result demonstrates that consecutive proline codons in a protein sequence might significantly slow-down ribosomal translation *in vivo*. The report of an elongation an factor, EF-P, that facilitates translation of consecutive proline residues [38] would suggest that the effects we see are due primarily to peptide bond formation and not context. This is consistent with the observation that levels of ribosomal stalling with the Pro-Pro inserts is far greater than that seen with individual Pro codons inserted at His4 or His5 (Figures 3 & 4) and supports the conclusion that formation of the Pro-Pro peptide bond is slower than formation of the peptide bond of proline to any other amino acid even in the presence of EF-P.

### A screen for hyper-slow translation codon pairs

The MacConkey-lactose indicator medium used in experiments presented in Figures 2 and 3 proved suitable for detecting a whole range of responses to apparent ribosome translation speeds. In some respects, however, this system is limited by its sensitivity, as relatively small changes in *his* expression levels can result in a red colony phenotype. We reasoned that a less sensitive indicator medium might be more suitable to identify codon combinations causing particularly strong stalling effects. When triphenyl-tetrazolium chloride-lactose (Tz-Lac) indicator medium is used, significantly higher β-gal activity is required to produce a Lac^+^ phenotype. For example, when the constructs in Figure 3 are scored for their appearance on Tz-Lac indicator plates, only the stop codon insertions at His5 are Lac^+^. The Pro-Pro codons at His4-His5 also give a strong Lac^+^ phenotype on Tz-Lac indicator medium, which is consistent with the high level of *hisD-lac* expression obtained with these constructs. We thus used Tz-Lac medium to quickly identify all the codon pairs at His4-His5 that would result in prolonged ribosomal stalling similar to that observed with stop codons.

The His4-His5 positions in the *his* leader were mutated with C/A/GNN x C/A/GNN in the *hisD-lac* background and plated on Tz-Lac indicator medium. Sixteen Tz-Lac^+^ colonies were purified and analyzed by DNA sequence analysis to determine what codon pairs resulted in high levels of *hisD-lac* expression due to loss of attenuation. The UNN codons were specifically avoided to eliminate the isolation of stop codons. The 16 translation-slow pairs identified in this screen are shown in Table 1.

**Table 1:** Codon pairs at His4-His5 that result in a Tz-Lac^+^ phenotype.

Isolate	Codon pair*	Amino acid pair
1	CGG-AGG (−9.0)	Arg-Arg
2	AGG-CGG (−4.6)	Arg-Arg
3	AAG-AGG (−9.1)	Lys-Arg
4	CCA-CCC (0)	Pro-Pro
5	CCG-CCA (0)	Pro-Pro
6	GTG-GGA (−3.6)	Val-Gly
7	CCT-CCG (0)	Pro-Pro
8	CCG-CCG (0)	Pro-Pro
9	CGA-CGG (−1.5)	Arg-Arg
10	CCT-CCT (0)	Pro-Pro
11	CGG-AGG (−9.0)	Arg-Arg
12	CCG-GGT (−3.2)	Pro-Gly
13	CAG-GAT (−3.7)	Gln-Asp
14	CCA-CCG (0)	Pro-Pro
15	CTG-AGG (−5.0)	Leu-Arg
16	CCG-GGG (−4.6)	Pro-Gly

*Calculated Anti-SD free energies [27] are shown in parentheses.

Of the 16 pairs sequenced, only two isolates (1 and 11) had the same codon pairs indicating that this screen was not saturating and did not identify all possible translation-slow pairs. Significantly, 6 of the 16 translation-slow pairs were Pro-Pro as predicted from the results described above. Also noteworthy is that four of the translation-slow pairs contained the rare Arg codon AGG. In the case of the pairs containing the rare AGG codon it seemed likely that tRNA limitation results in the translation-slow phenotype. However, with the recent determination that a Shine-Dalgarno sequence would result in ribosome stalling [27] we note that the codon pairs containing one or two Arg codons all have significant anti-Shine-Dalgarno free energies with the exception of the CGA-CGG pair, which does not include a rare Arg codon. Other codon pairs that were lacking Arg and were not di-proline pairs all showed significant anti-Shine-Dalgarno free energies, which could account for their translation-slow phenotype. These results demonstrate that the Tz-Lac screen can be used to identify codon pairs that result in high degree of ribosome stalling *in vivo*.

Since 6 of the 16 different pairs were Pro-Pro and there are 16 possible Pro-Pro pairs possible there should be 16/6×15 or roughly 40 possible codon pairs that are translation-slow similar to what was observed with a stop codon in the *his* leader peptide gene assay for the C/A/GNN x C/A/GNN substitutions at His4-His5. Since codons beginning with U were avoided in this screen, the 40 possible codon pairs represent about 75% (48/61×48/61) of all possible coding pairs. Thus, we predict about 50 total codon pairs that are as translation-slow as a stop codon in the His leader sequence should exist. A thorough search of all translation-slow pairs may allow for the identification of essential translation-slow pairs in the *Salmonella* genome. Replacing translation-slow pairs with translation-fast pairs (and vice-versa) followed by assay for protein production or activity to determine if a codon pair must stay translation-slow or translation-fast remains to be tested.

### Effects of Arg codons and Shine-Dalgarno-like sequences on the speed of ribosome translation

We next wanted to determine if the translation slow effects of rare Arg codons was due to their potential 16s anti-Shine-Dalgarno interacting activity, their limited charged tRNA levels or a combination of the two. There are 6 codons for the amino acid arginine (Arg): CGN and AGA/G. The tRNA species that read three of these codons: CGG, AGA and AGG, are present in low abundance and along with the CGA codon are all at low abundance [39]. The results presented in Figure 3 show the rare arginine codon AGG at position His5 had the strongest effect on translation of the *his* leader peptide gene. This would account for the difficultly in expressing proteins containing these rare codons and on the reduced speed of translating these codons in the *his* attenuation system. Note that a single tRNA species reads both the AGA and AGG codon while a second tRNA species reads AGG, yet AGA is translated more efficiently than AGG. We conclude that the AGA codon is better recognized than the AGG codon during translation. We were also curious about the effects of the CGU/C/A Arg codons at His5 on translation speed since there are four tRNA gene products that read these codons (*argQ*, *argV*, *argY* and *argZ*). All three CGU/C/A codons are read by two tRNA species that are expressed from a tRNA gene present in two copies (*argQ* = *argV* and *argY* = *argZ*) in the chromosome. We observed a significant difference in attenuation with CGC at His5 compared to CGU and CGA at His5, where the CGU and CGA codons both resulted in high levels of de-attenuation (Figure 3). It appeared that the CGC codon was better recognized than the CGU and CGA Arg codons during translation; however, results presented below suggest the CGU de-attenuation level might be due to a context effect with CAU(His) at His4 because placement of CGU at both His4 and His5 resulted in a strong level of attenuation (see below).

**Figure 7:**
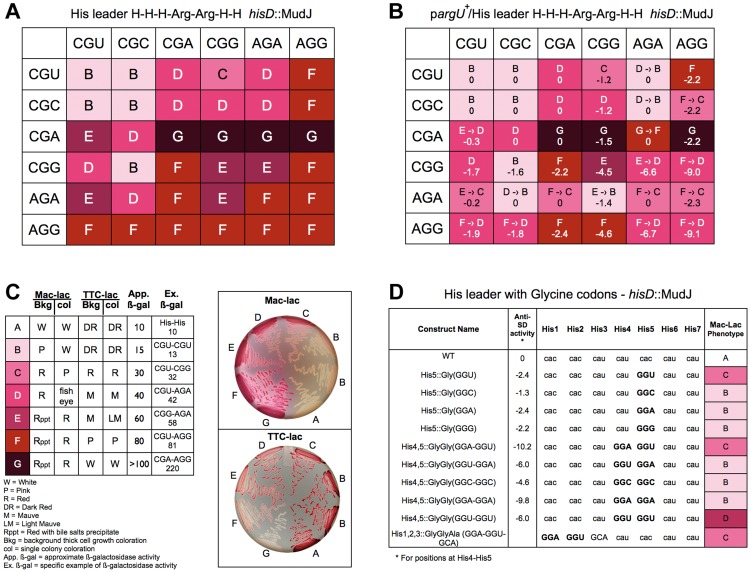
Expression of the *his* operon for specific arginine and glycine codons at the His leader on ribosome stalling. **A**. The levels of *hisD-lac* transcription for all strains with the His4-His5 codon positions in the His leader substituted with the 36 possible Arg-Arg codon pairs were determined by phenotypic assay on MacConkey-lactose (Mac-Lac) and tetrazolium-lactose (Tz-Lac) indicator medium. On Mac-Lac plates increasing levels of *lac* operon expression result in a darkening of red colony color transitioning from white to dark red while on Tz-Lac plates the reverse is true: on Tz-Lac plates, increased *lac* operon expression transitions from dark red to a white colony phenotype (see Fig. **7C**). By examining Lac morphologies in Mac-Lac and Tz-Lac indicator plates we were able to separate the His4-His5 Arg-Arg codon pair strains (labeled B–G), which are represented by the indicated color phenotypes where white represents the lowest level of *lac* operon expression; the transition from light pink to darker shades of red to dark violet at the highest level of *lac* operon expression. **B**. The effect of *argU* over-expression on *hisD-lac* transcription with each of the 36 possible Arg-Arg codon pairs placed at positions His4-His5 of the his leader peptide gene. The levels of *hisD-lac* expression is indicated by color shading (labeled B–F). A shift in *hisD-lac* expression is depicted by an arrow indicating the change in expression level from the parent strains in **A** that are lacking the *argU* over-expression plasmid. **C**. This figure indicates the color scheme used to determine that *hisD-lac* expression levels based on phenotype of thick background cells and individual colonies on Mac-Lac and Tz-Lac indicator plates. The wild-type strain with histidine codons at His4-His5 was used to indicate the expression level “A”. All of the Arg-Arg pairs at His4-His5 exhibited expression levels in the “B-G” range. The approximate β-galactosidase activity for each level is based on measured levels for the seven specific codon pairs at His4-His5 shown. **D**. The levels of *hisD-lac* transcription for strains with single glycine codons at His5 compared to (i) the wild-type all-His codons, (ii) to Gly-Gly codons GGA-GGU, GGU-GGA, GGC-GGC, GGA-GGA, GGU-GGU at His4-His5 and (iii) a perfect Shine-Dalgarno sequence at His1-His2, which includes the 5′-“G” nucleotide of a GCA Ala codon at His3. Levels of deattenuation were determined by phenotypic assay on MacConkey-lactose (Mac-Lac) and tetrazolium-lactose (Tz-Lac) indicator medium as described in Figure **7C**.

We decided to examine the effect of Arg codon pairs at His4 and His5 on *his* attenuation. If rare codons are slow to be translated due to tRNA limitation then all codon pairs with either AGA or AGG should show high levels of de-attenuation. If the CGC codon is translated more efficiently than the CGU or CGA codon then all codon pairs with CGC should exhibit lower levels of de-attenuation. However, the data presented in Figure 7 suggest that both CGU and CGC and are translated significantly faster than CGA.

All 36 possible Arg-Arg codons pairs were constructed at His4-His5 and their effect on loss of attenuation was determined by *hisD-lac* expression. As shown in Figure 7A, all Arg-Arg codon pairs with rare codons CGA, AGA and AGG showed high level of de-attenuation regardless of whether they were at the His4 or His5 positions. The levels of de-attenuation for each Arg-Arg codon pair were distinguished on MacConkey-lactose and triphenyl tetrazolium chloride-lactose indicator medium as shown in Figure 7B. For any Arg codon except AGG, pairing with a CGA codon showed the highest level of de-attenuation compared to when it was paired with any of the other 4 Arg codons. This is consistent with the idea that the tRNAs for CGU/C/A translates the CGA codon poorly compared to the other two codons and that the tRNA for AGA and AGG translates the AGA more efficiently than AGG. Interestingly, the CGU codon at His5 alone (Figure 3) showed the second highest level of stalling (excluding nonsense codons) after AGG; however, when the CAU His4 codon was replaced with either the CGU or CGC Arg codon the stalling phenotype was completely suppressed. This demonstrates a strong context effect of a 5′CAU (His) codon on translation of the CGU (Arg) codon.

The *argU* gene codes for the rare tRNA species that recognizes AGA and AGG. An *argU*-expression plasmid was introduced into the different strains with each of the 36 Arg-Arg codon pairs at His4-His5 of the *his* leader peptide gene. Figure 7C shows the effect of *argU* over-expression for each Arg-Arg pair at His4-His5. Also included in Figure 7C are the calculated anti-Shine-Dalgarno interacting free energies for each Arg-Arg pair. The *argU*-expression plasmid was also introduced into two control strains, one with the wild-type *his* leader, but deleted for the EF attenuator and one with a UAG stop codon at His5. In both cases β-gal activities increased 30% in the presence of the *argU*-expression plasmid indicating the background effect of *lacZ* gene expression from the *hisD-lac* operon reporter. While *argU* over-expression resulted in increased *his* operon transcription for 16 of the 20 Arg-Arg pairs with at least one AGG or AGA codon, the levels of de-attenuation remained high, which could be accounted to some degree by anti-SD interacting contributions with AGG- and AGA-containing codon pairs.

In order to clarify what extent 16S RNA-dependent ribosome stalling affects translation speed in the His leader peptide assay, we assayed tandem glycine codons including GGA-GGU, which is a perfect Shine-Dalgarno match (Figure 7D). Of the 61 single coding-codons placed at His5, GGU showed the 5th highest degree of de-attenuation based on MacConkey indicator phenotypes (Figure 3). If we compare the perfect Shine-Dalgarno match, GGA-GGU, at His4-His5 to the imperfect match with the same codon composition, GGU-GGA, we observe the same levels of de-attenuation seen with single GGU and GGA codons, respectively. This result suggests that the level of de-attenuation with either GGU or GGA at His5 is due to a context effect with the CAU(His) codon at His6. Duplicated Gly codons, GGC-GGC and GGA-GGA exhibited similar de-attenuation phenotypes as GGC and GGA alone at His5, while GGU-GGU showed stronger de-attenuation than GGU alone or as seen with the perfect Shine-Dalgarno match, GGA-GGU, at His4-His5. This suggests a context effect between successive GGU codons resulting in increased de-attenutation. Finally, since the Shine-Dalgarno sequence should have its effect about 9 nucleotides later in the translated sequence, we also placed a perfect Shine-Dalgarno sequence at His1-His2. His3 was changed to GCA(Ala) to complete the perfect Shine-Dalgarno sequence and GCA was observed to be translated fast in the His leader peptide system (Figure 3). Here we do see an affect on de-attenuation as compared to the wild-type with all His codons, but similar to GGU alone at His5 or GGA-GGU at His4-His5. We conclude that both context and anti-SD interacting activity contribute to translation speed.

## Discussion

In the decades of studies on the Central Dogma of Molecular Biology from DNA to RNA to protein [40], the process of translation remains elusive to simple rules. The triplet nature of the genetic code is the cornerstone of mRNA translation [41], yet the process appears more complex than originally deduced. The elucidation of the 3-dimensional structure of the ribosome by X-ray crystallography has been a major achievement in the last decade providing great insight into the detailed molecular workings of this machinery [42], which is believed to be a left-over from an RNA-dominated world. One major puzzle in the translation field is the contribution of codon context, which can have a profound effect on codon translation.

Degeneracy makes decoding far more complicated than if only one codon would code for a given amino acid. Not only are there multiple tRNAs for most amino acids, a given tRNA can exist in multiple copies. During the analyses of many mutant hunts this lab and many others has routinely discarded alleles resulting from silent mutations with the thought that there must be another mutation elsewhere in the genome that accounted for the mutant phenotype. We are now aware that codon context can play a profoundly determinant role in the translation of synonymous alleles. The recent discovery of the role of the Shine-Dalgarno sequence identity in the translation process [27] leads us to wonder what other factors remain to be discovered that are important in translation.

With this in mind we set to establish a system in which translation speed through a given codon or set of codons could be assayed *in vivo*. We sought an assay that could minimize the effects of protein and mRNA stability. We were aware that it would be essentially impossible to devise an *in vivo* system that was independent of mRNA secondary and tertiary structures. For our purposes, we chose the *his* leader peptide gene system from *Salmonella enterica*. In bacteria transcription and translation are coupled. The ability to transcribe the *his* biosynthetic operon structural genes depends directly on translation of the upstream 16 amino acid *his* leader peptide gene. The ability to continue transcription into the structural genes requires that the *his* leader peptide gene be translated and that translation stalls within seven consecutive histidine codons. The stalling of translation prevents formation of a strong attenuator encoded in the region between the *his* leader peptide gene and the first *his* operon structural gene. This system allowed us to measure effects of specific codons and codon-translation *in vivo* with a 30-fold range in readout of our assay. Furthermore, the addition of a *lac* operon reporter allowed for direct visual readout of differential effects on translation. We should clarify that our experimental system is a genetic test of translation speed and therefore the experimental readout is not explicitly a measure of translation rate.

We draw a number of important conclusions from our study. First, is that every base has the potential to matter in the process of translation. The data presented in Figure 3 demonstrate that silent mutations can dramatically affect translation speed. We can't rule out the formal possibility that the small effects of single base “silent” changes on the apparent translation speed *in vivo* are due to effects on mRNA secondary or tertiary structure. However, such effects of single base “silent” changes would also happen during translation of any mRNA sequence and their specific effect would likely depend on ribosome occupancy of a given mRNA. The less efficient a given mRNA is translated at a particular codon, we presume the more downstream bases would be unprotected by the bound ribosomes allowing base-specific effects on mRNA secondary and tertiary structures. Goodman et al. [43] have shown that rare codons are enriched at the N-terminus of genes, which can affect mRNA secondary structure to control overall gene expression. A second significant finding of this study was the ability to observe the importance of context in translation speed through codons (discussed below). Third, this work is consistent with previous study demonstrating that codon pairs with significant 16s anti-Shine-Dalgarno (anti-SD) interacting sequence can impair translation-speed. A fourth significant result of this study was the identification of di-Pro codon pairs as being among the slowest codon pairs translated *in* vivo. In a screen of more than 30,000 colonies with C/A/GNN x C/A/GNN substitutions at His4-His5, we have identified the 16 di-proline codon pairs as among the 50 slowest codon pairs of the 3,721 codon pairs not containing termination codons in *Salmonella*. Codon pairs that have significant Shine-Dalgano sequences including those with rare Arg codons are among these 50 slowest codon pairs.

One result that might have been expected was the effect of rare arginine codons on *in vivo* translation speed. The strongest effect of single, non-termination codons placed at His5 of the *his* leader peptide gene was observed with AGG (Arg). Recent work using ribosome profiling demonstrated that synonymous changes to a coding sequence that is more identical to the Shine-Dalgarno sequence results in translational pausing in *E. coli* [27]. Any codon pair that includes AGG is expected to have significant anti-SD interacting activity and could explain why it is a rare codon. Introduction of a plasmid that overexpresses the *argU* gene, which encodes the tRNA that reads AGG and AGA codons, did increase apparent translation speed *in vivo* for 16 out of the 20 Arg-Arg codon pairs containing an AGG or AGA codon based on changes in *hisD-lac* expression. However, even with the addition of the *argU* expression plasmid, translation speed of codon pairs with AGG remained slow, which could be accounted for by their anti-SD interacting activity. Other rare codons did not lead to slow translation under nutrient rich conditions, which is consistent to what was observed by the ribosome profiling method [27]. Finally, we specifically looked at the effect at glycine codon pairs including GGA-GGU, which is a perfect Shine-Dalgarno match. The perfect Shine-Dalgarno match sequence did showed reduced translation speed when placed early at the His1-His2 positions of the His leader peptide assay consistent with acting about 9 nucleotides upstream of the potential translational stall [27].

The *his* leader peptide gene system allowed us to measure a range of 5′- and 3′-context effects on the UCA codon over a 30-fold range. Of the 64 codon pairs with UCA at His4 or His5, 21 pairs showed differences in de-attenuation significant enough to be visualized on MacConkey-lactose indicator plates. These results reaffirm that context plays an important role in ribosome translation. The effects we observe of codon identity and context on apparent ribosome speed through the *his* leader positions His4-His5 can explain the mounting data demonstrating codon bias in a variety of systems. Selection based on codon bias is well established (reviewed by [44]). Codon pair biases are readily observed in both prokaryotes and eukaryotes [5]. In bacteria, codon bias correlates with generation time [45]. Highly expressed genes show strong codon biases [46]–[47]. The speed of translation by the ribosome are affected by codon context [48]. Genome-wide changes in codon pair biases attenuate poliovirus growth [49]. In addition to effects on gene expression, synonymous codon usage affects both RNA and protein structure [50]–[52].

In a seminal study Grosjean et al. [34] demonstrated the profound effect that base modifications can have on the stability of interacting tRNAs through anti-codon:anti-codon triplet hybridization. Using the translation "speedometer" assay of Curran & Yarus [29], Li et al. [53] demonstrated that the first step in the translation elongation cycle, the aminoacyl-tRNA selection step, was both positively and negatively affected by loss of tRNA modification in the anti-codon loop of specific tRNA species. We suspect that it is these tRNA modifications that contribute to the context effects we observe in our His leader peptide assay possibly by affecting the stacking energy of a given tRNA that is brought to the A-site by EF-Tu with a tRNA in the P-site, which could be context dependent and affect the translation speed through specific codon pairs.

Additionally, our results have implications for gene expression. Overall gene expression including the formation of a stable protein product is dependent on translation speed, which affects co-translational degradation [54]. Synonymous changes in actin isoforms having nearly identical amino acid sequences had a dramatic effect on co-translational degradation [52]. When expressing heterologous proteins in *E. coli* the gene of interest is often redesigned to have codon biases to fit with that observed in *E. coli*. It is generally assumed that codon usage and RNA secondary structure are the major factors affecting translation speed. Our data demonstrates that codon context is a major factor affecting the speed at which proteins are translated suggesting that codon context should always be considered in redesigning genes for *E. coli* expression systems. This was apparent when we looked at the effect of UCA(Ser)-UAU/C(Tyr) codon pair at His4-His5 in the His leader. The UCA(Ser)-UAU/C(Tyr) codon pair exhibited *his* operon de-attenuation levels similar to that obtained with stop codons at those positions, yet the reverse UAU/C(Tyr)-UCA(Ser) pair resulted in no significant effect on *his* operon de-attenuation. We were also surprised to find that the CGU codon at His5 of the *his* leader peptide gene resulted in a translation slow phenotype, yet CGU at both His4 and His5 was translation-fast. Our data supports a model where different tRNA species located at the ribosome affect the overall stacking energy that sensed as each charged tRNA enters the “A” site and this ultimately determines the effect of codon context on *in vivo* translation speed.

Changing codon pair biases on a genomic scale has proven an effective way to attenuate viruses that make effective live vaccines [49]. It is likely that many of the effects of codon pair bias-derived changes on gene expression are due to changes in translation speed of different proteins. Similar methods that include codon pair speeds in designing attenuated essential genes may aid in the design of live bacterial vaccines.

There is substantial data supporting a model that translation efficiency of a given gene is primarily determined early as the ribosome transitions from the initiation phase through early elongation phase [24]. Secondary mRNA structures play a prominent role in this early elongation phase [43]. Secondary structures are going to vary for each gene having neutral, enhancing or retarding effects on further translation elongation. We propose that the presence of translation-fast or translation-slow codons and codon pairs in the N-terminal regions of genes will have different effects on translation efficiencies depending of whether exposure of mRNA secondary structures enhance or retard translation. The translation slowest codon pairs include those with significant anti-SD interacting activities and successive proline codons. Base stacking energies between tRNAs and mRNA at the “A”, “P” and “E” sites provide a mechanism for context effects observed between codons affecting translation speed that do not include Pro-Pro codons or anti-SD interacting codon pairs.

One aspect that remains to be analyzed is the effect of tRNA modifications, which are believed to contribute to base stacking energies during translation, on translation speed. We plan to use the *his* leader peptide gene system to examine effects of tRNA modifications on *in vivo* translation speed. The modification to pseudouridine by the product of the *hisT* gene occurs in the anti-codon recognition loop of 15 of the 43 tRNA species depicted in Figure 3, and the histidine tRNA has two pseudouridine modifications at positions 38 and 39 in the anti-codon loop. For over 40 years, *Salmonella* strains defective in the *hisT* gene have been known to be de-attenuated in *his* operon transcription [55]. Thus, we believe the *his* leader peptide system will allow us to examine effects of tRNA modification on *in vivo* translation speed in future studies.

## Materials and Methods

### Bacterial strains, plasmids and media

All strains used in this study were derived from *Salmonella enterica* serovar Typhimurium wild-type strain LT2. Cells were cultured in Luria broth (LB: Bacto Tryptone 10 g/l, Bacto Yeast Extract 5 g/l, NaCl 5 g/l). Amresco agar (12 g/l) was used for preparation of solid medium. Antibiotics were added to LB at the final concentrations: 100 µg/ml sodium ampicillin for plasmid-carrying strains, 15 µg/ml tetracycline-HCl (Tc) or anhydrotetracycline (ATc) (1 µg/ml). L-arabinose was supplemented to 0.2% (w/v) as needed. The generalized transducing phage of *S. typhimurium* P22 *HT105*/*1 int-201* was used in all transductional crosses [56].

### Strain constructions

Targeted chromosomal mutagenesis was carried out via the *tetRA* insertion and replacement with the λ Red recombinase system as described [57]. All Primers were synthesized by Integrated DNA Technologies (Coralville, IA) and listed in Table S1. All PCR reactions were performed using a proof reading polymerase (Accuprime Pfx, In vitrogen or Phusion, Fermenta). Recombinant products mediated by λ-Red were PCR-checked using Taq DNA polymerase and further sequenced at the DNA Sequencing Core Facility at the University of Utah.

The PCR-generated fragment was electroporated into strain pKD46/*hisD9953*::MudJ, using tetracycline as a positive selection. Strain TH15761 (pKD46/*hisO10508*::*tetRA hisD6653*::MudJ) was used to generate *his* leader peptide gene targeted constructs.

Strain TH15759 contains a *tetRA* cassette between the 6^th^ and 7^th^ histidine codon of the *his* operon leader peptide gene and was constructed as follows: A PCR fragment containing a *tetRA* cassette with flanking 40 bp of homology to the *his* leader region before and after the 7^th^ histidine codon was generated using a strain containing a Tn*10d*Tc as a template and primers His7tetR and His7tetA. The PCR-generated fragment was electroporated into strain pKD46/*hisD9953*::MudJ, using the λ-red recombination technique [58], and tetracycline as a positive selection to generate the *hisO10508*::*tetRA* insertion mutant carrying the *tetRA* cassette inserted between codons 6 and 7 of the *his* operon leader peptide gene. Strain TH15761 (pKD46/*hisO10508*::*tetRA hisD6653*::MudJ) was used to generate *his* leader peptide gene targeted constructs.

### Replacement of the histidine codons of the *his* operon leader peptide by TAG stop codons

Primers hisOhis1stop, hisOGhis2stop, hisOGhis3stop, hisOGhis4stop, hisOGhis5stop, hisOGhis6stop and hisOGhis7stop were used with reverse primer hisOrev and genomic DNA from strain LT2 to generate 7 PCR fragments containing the TAG stop codon at each of the histidine codon positions of the *his* leader peptide gene. PCR reactions were cleaned by ethanol precipitation and resuspended in 20 µl water prior to electroporation. An overnight culture of strain TH15761 (pKD46/*hisO10508*::*tetRA hisD6653*::MudJ) was grown at 30°C in 2 ml of LB supplemented with ampicillin. A 25 ml LB-Ap-arabinose culture was inoculated with the above overnight culture and grown with shaking at 30°C until the culture had reached an OD_600_ of 0.6. The cells were washed twice in cold sterile water and resuspended to a final volume of 250 µl with water. Ethanol-precipitated PCR products were electroporated into 40 µl of washed cells, incubated at room temperature for 1 h, and spread on tetracycline-sensitive plates (Tc^S^) (1 µg/ml anhydrotetracycline, sigma). Tc^S^ recombinants were selected at 37°C and streaked at 42°C to eliminate the plasmid pKD46. Recombinants were checked for replacement of the *tetRA* cassette and loss of the pKD46 plasmid (Ap^S^), verified by PCR and sent for sequencing analysis.

### Randomization of the histidine codon 5 of the *his* leader peptide gene

Primer hisOGhis5NNNfwdfill was filled-in with primer His5contextfillinrev using Accuprime DNA polymerase. The DNA fragment was ethanol precipitated, resuspended in 10 ul Millipore water, electroporated into strain TH15761 and plated on tetracycline-sensitive plates (Tc^S^) plates at 37°C as described above. Recombinants were isolated, assessed on MacConkey-lactose indicator plates for Lac-phenotypic color variations, and a range of colored colony variants were sent for DNA sequence analysis in order to maximize potential different codon combinations isolated. Remaining strains not isolated were constructed individually to cover the 64 codon combinations using the same sequence primer as hisOGhis5NNNfwdfill, but with the specific codon instead of NNN.

### Randomization of histidine codons 4 or 5 of the *his* leader peptide gene paired with the UCA-serine codon

Primer NNNHis5TCAfwd and primer His4TCANNNfwd were filled-in with primer His5contextfillinrev using Accuprime DNA polymerase (In vitrogen). The procedure used to prepare these his4-5::NNN-TCA and his4-5::TCA-NNN strains was the same as that used for making the strains randomized at codon His5 of the *his* leader peptide gene. Again, remaining strains were constructed individually to cover the 64 codon combinations, with the same sequence primers NNNHis5TCAfwd and His4TCANNNfwd, but with the specific missing codon instead of NNN.

### Pro-Pro strains construction

Strains containing di-proline codons at the His4 and His5 positions of the *his* leader peptide gene were constructed using primer His4-5CNN-CNN by λ-Red recombination as described above. After electroporation and plating on Tc^S^ selection plates, the plates were replica printed onto MacConkey-lactose and tetrazolium-lactose indicator plates. White colonies on tetrazolium plates (Lac^+^) were isolated and sent for DNA sequence analysis. The remaining strains carrying di-proline codon pairs were made individually using the specific primer of the missing di-proline codon pair.

### Arg-Arg and Gly-Gly strain construction

Strains containing di-arginine codons at position His4 and His5 of the *his* leader peptide gene were constructed using the following primers: A-His4-5CGN-AG(AG), B-His4-5AG(AG)-CGN, C-His4-5CGN-CGN, D-His4-5AG(AG)-AG(AG). Each filled-in primer reaction (with His5contextfillinrev) was electroporated into strain TH15761, as described above. After electroporation and plating on Tc^S^ selection plates, the plates were replica printed onto MacConkey-lactose and tetrazolium-lactose indicator plates. White colonies on tetrazolium plates (Lac^+^) were isolated and the *his* leader peptide gene region sequenced. The remaining di-arginine strains not obtained were made individually using the specific primer of the missing di-arginine pair.

Strains containing di-glycine codons at position His1-His-2 and His4-His5 of the *his* leader peptide gene were constructed using the following primers: A-His4-5 Gly-Gly GGA-GGU, B-His4-5 Gly-Gly GGU-GGA, C-His4-5 Gly-Gly GGC-GGC, D-His4-5 Gly-Gly GGA-GGA, E-His4-5 Gly-Gly GGU-GGU, and F-His1,2-perfectSD. Each filled-in primer reaction (with His5contextfillinrev) was electroporated into strain TH15761, as described above.

### Replacement of the histidine promoter with P*_tetA_*

In order to assay effect of translation on *his* operon attenuation, the -10 through -35 *his* promoter region of selected strains was replaced by the promoter of the *tetA* gene, so that the *his* leader peptide could be transcribed by addition of ATc with or without added spectinomycin to inhibit translation. A *tetRA* cassette was first introduced by λ-Red replacing the -10 and -35 *his* promoter region using primers -35hispromtetR and -10hispromtetA. The *tetA* gene and ribosome binding site of *tetA* were then deleted from the *tetRA* cassette by λ-Red recombination in the above *tetRA* strain (via pKD46) using primers 10histetAhisPfwd and hisrevprom and genomic DNA of LT2 as template. This resulted in replacement of the *his* operon promoter with the Tc- or ATc-inducible the *tetA* gene promoter.

### Screen for translation-slowest codon pairs

Strains containing slow pairs at position His4 and His5 positions of the *his* leader peptide gene were constructed using primer His4-5 C/A/G NN by λ-Red recombination as described above. After electroporation and plating on anhydrotetracyline plates, the plates were replica printed onto MacConkey-lactose and tetrazolium-lactose indicator plates. White colonies on tetrazolium plates (Lac^+^) were isolated and sent for DNA sequence analysis. The T-N-N codons were specifically avoided to prevent isolation of stop codons. The goal was to use Tz-Lac screening to identify translation-slow codon pairs in the *his* leader system.

### β-Galactosidase assays

Thirty microliters of an overnight culture was subcultured into 3 ml of fresh LB medium. Tubes were incubated with shaking at 37°C until the contents reached a mid-log-phase density of OD 0.4. Cultures were put on ice, spun down, and resuspended in 3 ml of cold-buffered saline. Culture samples of 0.5 ml (diluted if necessary) were added to 0.55 ml of complete Z-buffer (Z-buffer plus 5 µl of 10% sodium dodecyl sulfate and 100 µl of chloroform) [59]. The assay was continued as described previously [59]. For each strain, assays were performed for three independent biological replicates.

### Real Time-PCR assays

RNA isolation was performed for three independent biological replicates using the RNeasy minikit (Qiagen). For removal of genomic DNA, RNA was treated with DNase I for 30 min at 37°C using a DNA-free RNA kit (Zymo Research) or on-column treatment was performed using kit 79254 (Qiagen). Subsequently, RNA samples were reverse-transcribed according to the RevertAid first-strand cDNA synthesis kit (Fermentas). Quantitative real-time PCRs were performed using the EvaGreen quantitative real-time PCR master mix (Bio-Rad) and primers hisG-multiRT-fw (gaa aac atg ccg att gat atc ctg) and hisG-multiRT-rv (agc acg tttt cgc cga taa tac) for *hisG*, rpoA-RT-fw (cgc cct gtt gac gat ctg g) and rpoA-RT-rv (ttt acc caa gtt agg cgt ctt aag) for *rpoA* and gyrB-RT-fw (ctg ctc aaa gag ctg gtg tat ca) and gyrB-RT-rv (agc gcg tta cag tct gct cat) for *gyrB*. Experiments were performed on a CFX96 real-time PCR instrument (Bio-Rad). Relative changes in mRNA levels were analyzed according to the Pfaffl method [60] and normalized against the transcript levels of the reference genes *rpoA* and *gyrB*.

## Supporting Information

Figure S1 **A**. β-Galactosidase activities for various codon pairs at the His4-His5 positions in the *his* leader sequence using *hisD-lac* reporter constructs. The activities represent the average of 3 or more independent assays. **B**. The β-galactosidase activities for the 16 possible Pro-Pro codon pairs at the His4-His5 positions in the *his* leader sequence using *hisD-lac* reporter constructs. The activities represent the average of 3 or more independent assays and were used to generate the data presented in Figure 6B.
(PDF)

Table S1 This table lists all primers used in this study as described in the Materials and Methods section. All sequences are written in a 5′ to 3′ direction.
(PDF)
