# Supplementary material for: The Effects of Codon Context on In Vivo Translation Speed
Source: PLoS Genet. 2014 Jun 5;10(6):e1004392. doi: 10.1371/journal.pgen.1004392 (PMC4046918; doi:10.1371/journal.pgen.1004392)
Supplement: Figure S1 — A. β-Galactosidase activities for various codon pairs at the His4-His5 positions in the his leader sequence using hisD-lac reporter constructs. The activities represent the average of 3 or more independent assays. B. The β-galactosidase activities for the 16 possible Pro-Pro codon pairs at the His4-His5 positions in the his leader sequence using hisD-lac reporter constructs. The activities represent the average of 3 or more independent assays and were used to generate the data presented in Figure 6B. (PDF) [file pgen.1004392.s001.pdf]

Supporting Information Figure S1.

A.

Second position of codon

First position of codon (5' end)	U	C	A	G	Third position of codon (3' end)
U	24.3 ± 1.7	21.0 ± 2.7	31.0 ± 3.2	48.1 ± 4.5	U
	22.5 ± 1.1	22.7 ± 3.5	24.8 ± 3.1	21.8 ± 0.8	C
	18.1 ± 0.9	15.1 ± 1.4	276 ± 20	259 ± 12	A
	16.9 ± 0.5	17.0 ± 0.2	292 ± 28	26.2 ± 4.7	G
C	25.3 ± 0.8	27.4 ± 3.7	18.4 ± 1.0	87.1 ± 5.1	U
	23.6 ± 1.7	36.5 ± 1.6	11.4 ± 2.1	19.0 ± 1.0	C
	17.7 ± 1.3	18.2 ± 1.8	16.5 ± 1.3	80.1 ± 12	A
	10.7 ± 0.5	16.6 ± 1.8	11.3 ± 1.4	45.5 ± 1.0	G
A	19.9 ± 1.0	11.8 ± 1.6	20.7 ± 2.8	74.1 ± 3.6	U
	17.3 ± 1.5	12.9 ± 2.4	14.9 ± 3.3	15.3 ± 1.9	C
	31.7 ± 1.4	9.6 ± 1.9	14.0 ± 2.0	55.0 ± 3.6	A
	11.5 ± 3.6	9.1 ± 2.3	13.3 ± 2.1	102 ± 15	G
G	20.0 ± 1.3	12.4 ± 1.5	25.7 ± 1.3	56.9 ± 1.5	U
	19.5 ± 0.7	10.7 ± 1.0	16.5 ± 1.2	18.6 ± 0.8	C
	12.2 ± 1.4	8.0 ± 0.9	19.0 ± 1.1	23.0 ± 0.4	A
	14.2 ± 2.3	8.1 ± 0.8	21.5 ± 1.5	21.8 ± 0.9	G

B.

His4 \ His5	CCU	CCC	CCA	CCG	CAC
CCU	354±24	340±5	350±16	344±5	—
CCC	363±6	372±2	344±21	341±21	—
CCA	355±13	345±7	348±16	339±26	—
CCG	318±10	325±3	262±13	266±1	—
CAU	—	—	—	—	11.0±0.3
