# Supplementary material for: The Effects of Codon Context on In Vivo Translation Speed
Source: PLoS Genet. 2014 Jun 5;10(6):e1004392. doi: 10.1371/journal.pgen.1004392 (PMC4046918; doi:10.1371/journal.pgen.1004392)
Supplement: Table S1 — This table lists all primers used in this study as described in the Materials and Methods section. All sequences are written in a 5′ to 3′ direction. (PDF) [file pgen.1004392.s002.pdf]

Supporting information Table S1- List of primers

Name	Sequence
His7tetR	tat gac acg cgt tca att taa aca cca cca tca tca cca ttt aag acc cac ttt cac att
His7tetA	ctg aat gtc ttc cag cac aca tcg cct gaa aga cta gtc agg cta agc act tgt ctc ctg
hisOhis1stop	gca ttc atc gga att ttt atg aca cgc gtt caa ttt aaa **tag** cac cat cat cac cat cat
hisOGhis2stop	ttc atc gga att ttt atg aca cgc gtt caa ttt aaa cac **tag** cat cat cac cat cat cct
hisOGhis3stop	atc gga att ttt atg aca cgt gtt caa ttt aaa cac cac **tag** cat cac cat cat cct gac
hisOGhis4stop	gga att ttt atg aca cgc gtt caa ttt aaa cac cac cat **tag** cac cat cat cct gac tag
hisOGhis5stop	att ttt atg aca cgc gtt caa ttt aaa cac cac cat cat **tag** cat cat cct gac tag tct
hisOGhis6stop	ttt atg aca cgc gtt caa ttt aaa cac cac cat cat cac **tag** cat cct gac tag tct ttc
hisOGhis7stop	atg aca cgc gtt caa ttt aaa cac cac cat cat cac cat **tag** cct gac tag tct ttc agg
hisOrev	tct gaa ccg gtc tgt atc
hisOGhis5NNNfwdfill	c gga att ttt atg aca cgc gtt caa ttt aaa cac cac cat cat **nnn** cat cat cct gac tag tct ttc agg cga tgt gtg ctg gaa gac a
His5contextfillinrev	tgtcttccagcacacatcg
NNNHis5TCAfwd	c gga att ttt atg aca cgc gtt caa ttt aaa cac cac cat **nnn tca** cat cat cct gac tag tct ttc agg cga tgt gtg ctg gaa gac a
His4TCANNNfwd	c gga att ttt at g aca cgc gtt caa ttt aaa cac cac cat **tca nnn** cat cat cct gac tag tct ttc agg cga tgt gtg ctg gaa gac a
His4-5CNN-CNN	c gga att ttt atg aca cgc gtt caa ttt aaa cac cac cat **ccn ccn** cat cat cct gac tag tct ttc agg cga tgt gtg ctg gaa gac a
His4-5CGN-AG(AG)	cgg aat ttt tat gac acg cgt tca att taa a cac cac cat CGN AGR cat ca t cct gacta gtc ttt cag gcg atg tgt gct gga aga ca
His4-5AG(AG)-CGN	cgg aat ttt tat gac acg cgt tca att taa a cac cac cat AGR CGN cat ca t cct gacta gtc ttt cag gcg atg tgt gct gga aga ca
His4-5CGN-CGN	cgg aat ttt tat gac acg cgt tca att taa a cac cac cat CGN CGNcat ca t cct gacta gtc ttt cag gcg atg tgt gct gga aga ca
His4-5AG(AG)-AG(AG)	cgg aat ttt tat gac acg cgt tca att taa a cac cac cat AGR AGR cat ca t cct gacta gtc ttt cag gcg atg tgt gct gga aga ca
-35hispromtetR	act gac aat tca tac tat aaa tgc gaa atg aaa aaa gcg c TTA AGA CCC ACT TTC ACA TT
-10hispromtetA	tca taa aaa ttc cga tga atg ctt att cat ttg ata cct ttt CTA AGC ACT TGT CTC CTG
10histetAhisPfwd	att ttt gtt gac act cta tca ttg ata gag tta ttt tac ca GGTA TCA AAT GAA TAA
hisrevprom	gtg ttt aaa ttg aac gcg tg
His4-5 C/A/G NN	c gga att ttt atg aca cgc gtt caa ttt aaa cac cac cat **VNN VNN** cat cat cct gac tag tct ttc agg cga tgt gtg ctg gaa gac a
His4,5-GlyGly GGA-GGU	cgg aat ttt tat gac acg cgt tca att taaa cac cac cat **gga ggt** cat cat cct gac tag tct ttc agg cga tgt gtg ctg gaa gac a
His4,5-GlyGly GGU-GGA	cgg aat ttt tat gac acg cgt tca att taaa cac cac cat **ggt gga** cat cat cct gac tag tct ttc agg cga tgt gtg ctg gaa g ac a
His4,5-GlyGly GGC-GGC	cgg aat ttt tat gac acg cgt tca att taaa cac cac cat **ggc ggc** cat cat cct gac tag tct ttc agg cga tgt gtg ctg gaa g ac a
His4,5-GlyGly GGA-GGA	cgg aat ttt tat gac acg cgt tca att taaa cac cac cat **gga gga** cat cat cct gac tag tct ttc agg cga tgt gtg ctg gaa g ac a

Supporting information Table S1- List of primers, *continued*

Name	Sequence
His4,5-GlyGly GGU-GGU	cgg aat ttt tat gac acg cgt tca att taaa cac cac cat **ggt ggt** cat cat cct gac tag tct ttc agg cga tgt gtg ctg gaa g ac a
His1,2-perfectSD	a gca ttcat cgg aat ttt tat gac acg cgt tca att taa a **gga ggu gca** cat cac cat cat cct gac tag tct ttc agg cga tgt gtg ctg gaa gac a
hisG-multiRT-fw	gaa aac atg ccg att gat atc ctg
hisG-multiRT-rv	agc acg tttt cgc cga taa tac
rpoA-RT-fw	cgc cct gtt gac gat ctg g
rpoA-RT-rv	ttt acc caa gtt agg cgt ctt aag
gyrB-RT-fw	ctg ctc aaa gag ctg gtg tat ca
gyrB-RT-rv	agc gcg tta cag tct gct cat
